# FME-24: A film, music, and emotion dataset

**DOI:** 10.1007/s13735-026-00404-z

**Published:** 2026-06-02

**Authors:** Ruby Olivia Nagano Crocker, György Fazekas

**Affiliations:** Centre for Digital Music, Queen Mary University of London, Mile End Road, London, E1 4NS UK

**Keywords:** Music Emotion, Film Music, Temporal Music Analysis, Film Emotion, Datasets, Emotion Annotation, Valence-Arousal, Music Datasets, Multimodal Analysis

## Abstract

This paper introduces an updated, publicly accessible version of the Film, Music and Emotion Dataset (FME-24), designed to examine how perceived emotion in film music evolves over time. It provides a comprehensive introduction to the dataset and explores its potential applications across music information retrieval (MIR), psychology, and AI-training contexts. The FME-24 dataset utilises film’s immersive qualities to study emotional perception in a naturalistic yet controlled setting. It contains data from 275 film scores spanning the past two decades, including experimental and mainstream works. The dataset integrates high-quality film compositions with time-stamped valence-arousal (V-A) annotations, emotion sentences, familiarity ratings, and detailed metadata. 98 Participants contributed to annotating these temporal emotion features. For each time-stamped point, a two-second audio segment was analysed, and 78 features were extracted, including low-level timbral descriptors (MFCC statistics, spectral centroid), rhythmic descriptors (onset density, tempo), and higher-level psychoacoustic and tonal features (inharmonicity, roughness, chord transitions, tonal entropy). Although full audio files are unavailable due to licensing, reproducibility is ensured via ISRC codes, precise segment timings, and open access to all metadata and feature files in CSV format. The paper details the dataset’s structure, annotation, and feature-extraction procedures, highlighting applications in computational and perceptual research and laying a foundation for future studies on emotion, perception, and narrative in film music.

## Introduction

The FME-24 dataset was created to explore how listeners perceive emotional changes in film music over time. Composers intentionally use musical techniques to shape emotion, this dataset focuses on the perceptual side: how those compositional choices are perceived by listeners. This question arises from both scientific curiosity and recognition of the unique expressive role of film music [1]. Unlike music written for stand-alone listening, where the primary goals are entertainment or artistic expression, film music is composed to provide narrative, guide perception, and support visual storytelling [2]. Film scores are temporally bound to scene changes, character arcs, and emotional transitions.

While advances in computational methods continue to drive progress in Music Information Retrieval (MIR), the success of emotion-based analysis depends on high-quality, task-specific datasets. In film music, where emotional expression is nuanced and context-driven, such datasets are scarce, particularly those capturing the temporal evolution of emotion. FME-24 addresses this gap by providing a systematic, time-resolved resource of contemporary film music excerpts with continuous arousal-valence (V-A) annotations. Participants indicated perceived emotional changes by pressing a button whenever they experienced a shift in emotion while listening to each excerpt. A button-press paradigm was used because it captures emotional shifts in real time with minimal disruption, offering greater temporal precision and lower annotation fatigue than post-hoc labelling. Each button press marked a time-stamped point, and for every such point, two-second audio segments were extracted, See Section 6. From these segments, 78 musical features were computed, capturing aspects of frequency, key, timbre, texture, and other relevant musical qualities. The selected features come from well-validated MIR toolkits and algorithms that are widely used in affective computing and music emotion recognition (MER) research, ensuring that the descriptors reflect acoustic and musical dimensions known to contribute to emotional perception [3, 4]. This approach provides temporal mapping between musical structure and perceived emotional change. Importantly, the dataset focuses solely on audio, without visual context, allowing researchers to study how musical structure alone conveys and evolves emotional meaning while retaining the temporal dynamics inherent to narrative film scoring.

This paper acts as a usage guide, detailing dataset structure, annotation methodology and feature extraction processes. This work establishes a foundation for future studies on emotion perception in narrative-based music.

## Related work

Film offers an immersive and emotionally rich medium for studying human affective response, offering realism without the ethical or practical constraints of more artificial laboratory settings. Within this context, music functions as a carrier of unseen emotion, communicating nuance, tension, and narrative intent that may not be visually explicit [5]. Over the past century, film scoring has developed into a sophisticated emotional language; one capable of directing audience interpretation and heightening engagement. Film music now uses an increasingly diverse range of compositional styles and techniques, extending beyond traditional orchestral scoring, yet continues to evoke consistent emotional responses in audiences [1]. Selective musical cues can even outweigh visual information in shaping emotional meaning and perceived atmosphere [6].

Existing music emotion datasets vary in size, granularity, and methodology. FME-24 complements these resources by focusing specifically on contemporary film scores with precise temporal annotations. Modern film scoring has expanded beyond purely orchestral styles, incorporating electronic elements, sound design, and unconventional instrumentation [7], expanding the expressive palette available to composers. Due to this diversity, emotional content in modern film music can shift rapidly in terms of texture, intensity, and atmosphere. As a result, high-resolution annotation is essential to accurately capture these dynamic emotional changes. Contemporary scores matter because they reflect modern compositional styles, production techniques, and evolving emotional norms in film scoring, offering insights that older datasets may miss. Earlier publications on FME-24 described the annotation interface and initial analyses [8]; these are expanded on to provide a usage guide for researchers in MIR, psychology, and AI-driven music studies. Table 1 situates FME-24 among existing datasets, highlighting its unique combination of contemporary film scores, continuous emotion annotations, and rich metadata.

**Table 1:** Overview of emotion-labelled music datasets.

**Dataset**	**Year**	**#Clips**	**Ann.Type**	**Annot.**	**Length**	**Feat.**
Soundtracks [9]	2011	470	V+A & Cat	116	15-30s	8 (mood)
Moodo [10]	2016	200	V+A	741	15s	n/a
DEAM [11]	2017	1802	V+A	10/excerpt	45 sec	n/a
PMEmo [12]	2018	794	V+A	457	various	260
VGMIDI [13]	2019	200	V+A	30/excerpt	26s-3m	n/a
Jamendo [14]	2019	18486	Mood tags	n/a	$\ge 30$s	56 (mood)
CCMED [15]	2020	800	V+A	989	8-10s	305
MERP [16]	2022	54	V+A	277	2m52s	260
**FME-24**	2024	201	V+A + Cat	98	15s	78
Emotify+ [17]	2025	400	10 Cat	181	60s	15

### Emotion modelling in music

**Fig. 1:**
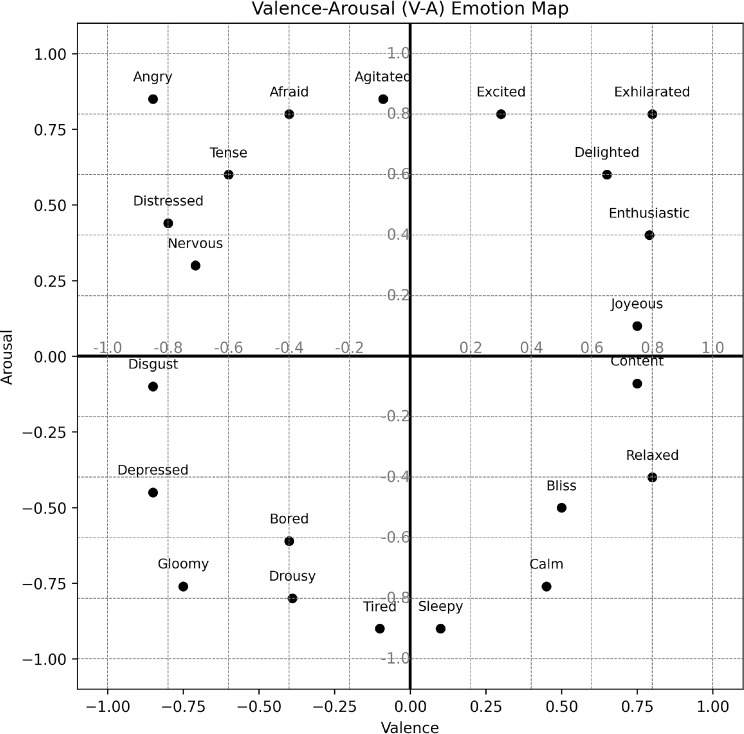
Russell’s Circumplex model of emotion in valence-arousal space [22].

Two dominant frameworks are commonly used to represent emotion in music: discrete emotion models and dimensional models. Discrete models, such as Ekman’s basic emotions, categorize affect into distinct states like happiness, sadness, anger, fear, surprise, and tenderness [18]. These have been widely applied in music emotion studies to investigate how specific musical features evoke particular emotional responses. [9, 19–21]. In contrast, dimensional models conceptualise emotion as a point in a continuous space, most commonly the V-A space as proposed by Russell [22] (see Figure 1). Here, valence represents the positivity or negativity of emotion, while arousal denotes its intensity or energy. This model is especially suitable for capturing emotional dynamics over time in music, which has led to its adoption in continuous annotation tasks in datasets like Database for Emotional Analysis of Music (*DEAM*) and Music Emotion Recognition with Profile information dataset (*MERP*).

The *Soundtracks* dataset [9] includes 360 royalty-free film music excerpts composed between 1976-2006. The use of royalty-free music minimized the influence of prior associations, focusing on the emotional content of the music. The dataset lacks continuous emotion annotations and focuses on older compositions, limiting its relevance for contemporary film scoring. The *MERP* dataset provides dynamic V-A annotations for 54 full-length songs, with demographic and musical background data from 277 participants. Although many datasets use royalty-free music to ensure public access, those using copyrighted film music typically release only derived data due to licensing restrictions.

### User-centric annotation design

User-centric item characteristics (UCICs), such as, perceived emotion and aesthetic appreciation, capture how individuals uniquely experience music. These personal and context-driven responses are central to MER, where emotional interpretation varies widely. Incorporating UCICs into annotation design allows researchers to create more personalized, accurate, and inclusive datasets [23]. The FME-24 dataset follows this approach by using free-text inputs and continuous V-A graph plotting, allowing annotators to express nuanced, unconstrained emotional responses rather than forcing them into fixed categories, See Section 3.

Listeners often describe music using varied, non-standard emotional terms, such as “dreamy” or “festive”, that differ from basic emotion labels like “happy” or “sad” and go beyond traditional V-A models. This highlights the diverse ways people express musical emotion, and allowing free-text responses supports more personalized and accurate annotations [24].

## Dataset overview

The FME-24 dataset (Film Music & Emotion - 2024) is a curated collection of 422 film music excerpts from 275 tracks, annotated with emotional responses from 98 participants. It includes a wide range of genres and soundtrack styles, drawn from films released between 2005 and 2023. An interactive online tool allowed participants to add continuous, real-time emotional annotations, where they could add, move, and resize data points to reflect their evolving feelings throughout a track. Each annotation point was automatically set to a 0.2-second duration, a timescale informed by research on human emotion perception in music [25], which was short enough to capture fine-grained changes, yet long enough to remain perceptually meaningful. This design allowed participants to provide as many or as few points as needed. After data annotation from participants, 178 excerpts were removed because they did not receive valid annotations or showed issues such as missing timestamps, incomplete V-A values, or participant-flagged errors (e.g., accidental markers or notes like “ignore this” or if all annotations by a participant were 0,0 for V-A). The released version of FME-24 therefore contains 422 annotated excerpts. Most excerpts received 2-3 annotations from each participant, although some had as many as 13. This meant overall in the dataset there were 1,784 individual data points of perceived change. Each of the 1,784 annotations is described by 78 temporal audio features extracted from librosa and essentia libraries.

Preliminary analyses of the free-text emotion annotations revealed considerable variability in expression. To enable analysis, sentences were preprocessed through lemmatization, mapping to V-A values and accounting for temporal cues. These processed sentences were then embedded and clustered, allowing them to be aligned with the continuous V-A values and represented as emotion category labels derived from participants’ subjective descriptions.

### Data layout

The structure of the main FME-24 CSV file is summarized in Table 2 (librosa_features_full_fme_dataset _MARCH_2026_NC.csv). Columns A to G contain participant IDs, audio file paths, annotation timestamps, emotion sentences, familiarity ratings, and valence-arousal coordinates. Columns H to M include imputed timestamps, annotation order, numbering, and start-end times for feature extraction, primarily used for data setup. Columns N to CM store the extracted audio features. Metadata for the audio files is provided in ‘film-emotion-music-datasheet.csv’, and participant demographics are in ‘fme-demographics-music-sophistication.csv’. The primary focus of the dataset is the continuous valence-arousal coordinates for regression analyses. The V-A coordinates range from -1 to +1. The emotion sentences enable secondary categorical analyses through semantic clustering. Preliminary clusters, using an optimal number of nine clusters, are provided in ‘fme-24-features-emotions-after.csv’ and ‘fme-24-features-emotions-before.csv’. Regarding sample rate, 67 out of 275 audio files were at 48kHz and the remaining files were at 44.1kHz.

**Table 2:** Overview of the FME-24 dataset - Main CSV Data Layout

**A**	**B**	**C**	**D**	**E**	**F,G**	**H–M**	**N–CM**
Part.ID	FileName	TimeStamp	Sentence	Familiar	V-A	Time-Start/End	Features
_0sca4...	thehunt...	2.56	I felt...	3	0.71, 0.16	1.56,3.56	rms,zcr...

**Fig. 2:**
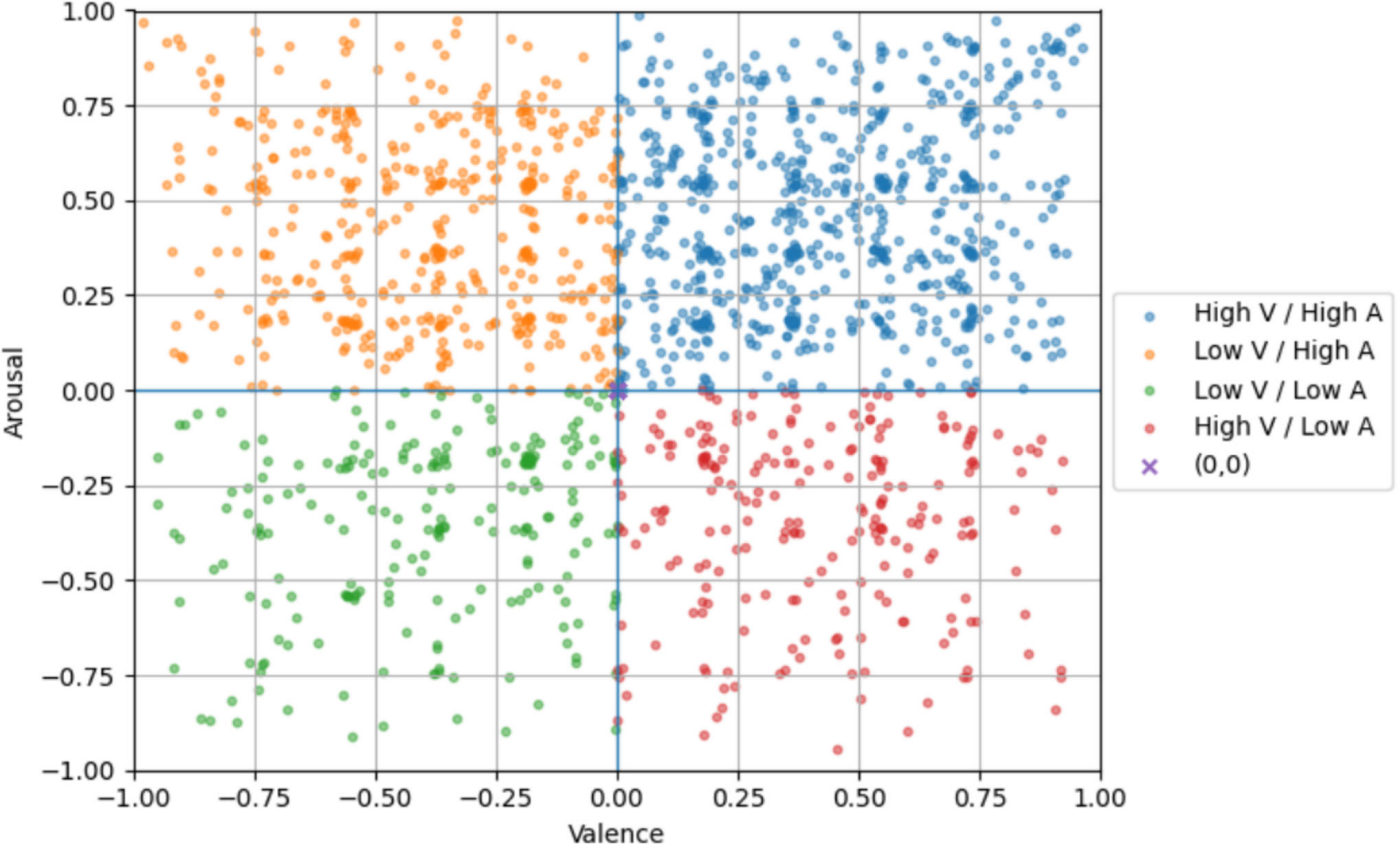
Valence-Arousal Coordinates Distribution in FME-24.

Valence-arousal coordinates were distributed across all four quadrants, with 681 coordinates in the high-valence/high-arousal quadrant, 449 in low-val/high-aro, 226 in low-val/low-aro, and 230 in high-val/low-aro, along with 187 points at the origin (0,0). This distribution shows broad coverage of the A-V space, with a tendency toward higher arousal regions, suggesting that the dataset captures a diverse range of emotional responses. The distribution of coordinates shows that participants mostly seem to avoid extreme values (±1), with valence and arousal that cover the full normalised range (valence: mean = 0.07, SD = 0.45, range = $-0.98$ to 0.96; arousal: mean = 0.19, SD = 0.42, range = $-0.95$ to 0.99). This pattern can be seen in Figure 2, where each quadrant is colour coded, showing a broad but centrally clustered distribution of responses in the V-A space.

### Selection criteria and content

Award-winning tracks were selected from major international film festivals and award ceremonies. In this context, award-winning refers to films that received recognised music-related awards across these institutions. This approach was preferred over random or stratified sampling because award-recognised scores generally have high production quality, clear emotional aims, and well-defined musical structure, making them easier to annotate reliably. This selection does, however, influence the dataset: although award-winning scores cover many styles, they may under-represent niche, experimental, or low-budget scoring, introducing some selection bias. Even so, the chosen tracks offer a consistent and emotionally rich basis for studying contemporary film-music perception.

Within those films, excerpts were chosen with an emphasis on scenes containing emotionally significant moments. These were defined as moments in the film where the music aligns with a clear narrative shift, character development, or change in mood. To mitigate individual bias, recommendations were sought from a small group of six film and music students and professionals actively engaged in film scoring or music supervision. This approach was intended to reduce the influence of any single individual’s judgment, though some subjectivity remains inherent in identifying emotionally significant moments. Fully random excerpt selection was avoided, as it risked including sections with minimal musical or emotional content, which would undermine the dataset’s purpose of capturing meaningful emotional changes.

Due to availability constraints, most selected films are European. Practical considerations such as the presence of ISRC codes also influenced which tracks were included, introducing a potential geographic bias. The final dataset prioritizes emotional diversity and includes high-quality film music over royalty-free sources.

The dataset includes 21 core film genres, with 72 unique genre labels due to IMDb’s multi-tagging system. This results in substantial overlap, with some genres (particularly *Drama*) frequently appearing in combination with others (e.g., *Drama, War*). At this stage, the full set of unique IMDb labels was kept to avoid additional subjective choices about which genre should take precedence when multiple tags are present. Genre categorisation might be explored in later analytical stages (predictive modelling), but it is intentionally deferred to preserve granularity in the initial dataset release.

FME-24 includes music from 196 composers/artists and consists mainly of original scores (213 tracks), alongside a smaller set of compilation soundtrack pieces (52 tracks) and 10 mixed tracks. Compilation tracks were intentionally included to examine whether pre-existing or stylistically different music evokes distinct affective responses compared with purpose-written film scores. This reflects current scoring practices, where filmmakers often use contrasting or recognizable songs to shape mood or narrative. Their status is clearly recorded in the metadata. In analysis, users may treat these tracks separately (example: by modelling original and compiled music independently) or include them with the understanding that participants’ responses may reflect recognition or genre associations. The dataset supports either approach.

## Annotation methodology

**Fig. 3:**
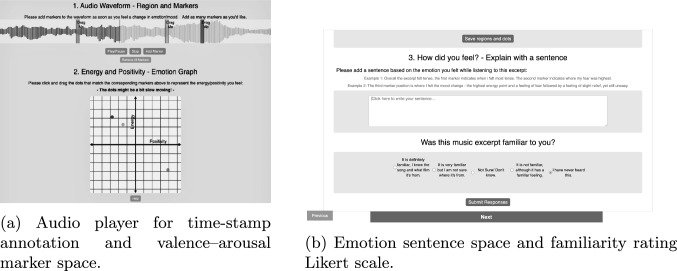
Online annotation tool used to collect emotion responses.

To collect emotion annotations, an interactive online tool was developed, See Figure 3. Before starting the annotation task, Participants first completed a demographic questionnaire and musical background survey, See section 4.2. They then viewed a tutorial to check volume levels and practice using the V-A tool before beginning the main task.

Each participant annotated 10 excerpts, each 15 seconds long, presented one per page without film visuals. Excerpts were initially drawn from a pool of 604 clips (two excerpts per 302 film tracks), but due to incomplete annotations, the final dataset included 422 excerpts from 275 tracks. The 15-second excerpt length and 10-excerpt limit per participant balanced the need to capture meaningful emotional responses while keeping the task manageable and reducing fatigue. Participants could stop at any time and all completed annotations would be saved. The clips were presented as audio-only waveforms, to isolate the emotional impact of the music itself [1, 26]. This dataset was initially created with the intention to study the emotional impact of narrative-based music in isolation, rather than interactions with film imagery.

**Fig. 4:**
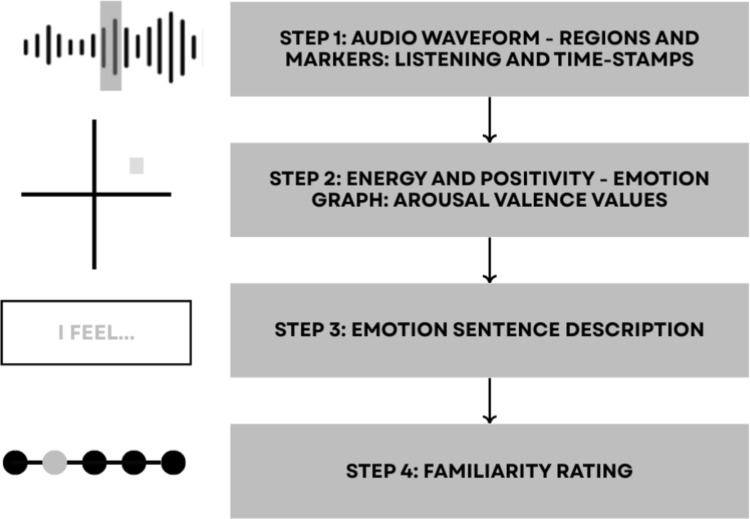
Flowchart of the annotation tool.

The embedded music player featured controls to play, pause, stop, add markers, adjust markers and remove all markers. This meant participants could re-listen when annotating, to readjust their markers. The flowchart of the annotation workflow is shown in Figure 4. The tool was created online where participants could access it via a website link, participants were encouraged to complete it alone, with high-quality headphones or speakers.

1. **Identify Emotional Shifts** Participants listened to an excerpt and clicked to place a marker the moment they felt a change in emotion. These markers could be moved to reflect participants’ accurate timings. Each marker was timestamped with a default window of ±0.2 seconds, which participants could manually adjust.
2. **Place Emotion on Valence-Arousal Graph** A coloured dot representing the marker appeared on a 2D V-A graph, positioned at the origin (0,0). The dot matched the colour of the corresponding marker on the audio waveform above, visually linking the two. Participants could move the dot to reflect the emotion they experienced in terms of valence and arousal. An optional ’help’ button displayed emotion adjectives on the graph to assist participants with interpretation. The placement of these adjectives was based on established V-A research [18, 22].
3. **Describe the Emotion** Participants then wrote a short“emotion sentence”to describe what they felt and explain marker positions. This was a free-text input and could be as concise or detailed as desired. It also served as a contingency check to ensure participants had engaged meaningfully with the annotation interface.
4. **Familiarity Rating** Finally, participants rated how familiar they were with the music excerpt on a Likert scale. Familiarity can significantly influence emotional response, through mechanisms such as, recognition, memory, and preference formation [27].

All clips were treated as beginning from a neutral affective baseline (0,0) for analytical purposes, as participants were not explicitly instructed to provide an initial emotional state. Given the short duration of the stimuli (approximately 15 seconds) and the focus on perceived changes in emotion over time, some participants reported one or two dominant emotions rather than continuously updating emotion changes.

To increase engagement in what is otherwise a time-intensive task, the study included a small gamified incentive: after completing all 10 annotations, participants received a personalised “music personality” profile. Gamification has been shown to improve completion rates in online studies [28]. Although dropout rates were not formally analysed, many participants reported the incentive was motivating and positively received.

### Participants

Participants were recruited via academic mailing lists and social media, targeting individuals likely to be interested in the study and motivated to complete the task. A total of 185 individuals initially took part in the annotation study. Data quality was ensured through a combination of automated checks and manual review. Automated filters removed submissions lacking essential information (e.g., missing timestamps, missing V-A coordinates, or absent track identifiers). Manual screening excluded low-engagement cases, such as participants who never moved the V-A cursor or whose text comments were unrelated to emotional perception. After these steps, 98 participants remained. Although a larger sample would be ideal for machine learning applications, the time-intensive nature of continuous emotional annotation constrained recruitment.

Professional training or familiarity with film music was not required as the study aimed to capture general audience responses rather than expert-only perspectives. However, participants who reported being emotionally responsive to music tended to provide nuanced and consistent emotional judgments, enhancing reliability [29]. Attention checks confirmed meaningful engagement. Annotations from musicians and non-musicians showed no significant differences; 57 Participants identified as musicians to some degree, while 32 identified more closely to non-musicians, and 9 were not sure, See Figure 6b.

**Fig. 5:**
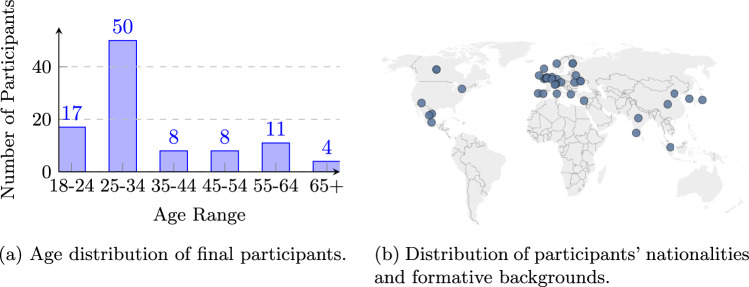
Participant demographics: (a) age distribution; (b) nationalities and formative backgrounds.

The age distribution of the final participants (98) was biased toward younger adults, with the largest group aged 25-34 (50), See Figure (Figure 5). The younger age profile might influence emotional annotation patterns (e.g., musical-style preference, valence/arousal perception, familiarity effects). This age profile likely reflects the recruitment platforms used.

Alongside age, participants provided information on nationality, formative country, gender, and occupation. Most were British (59) and from Europe (79), with smaller numbers from North America (10) and Asia (8), 2 participants left it blank. Formative country largely aligned with nationality, with 63 participants spending early years in the UK; when participants listed multiple countries, formative country was used for analyses (e.g., “Mexico and Canada”, “Mexican” ). This produced a “long tail”distribution, with the UK heavily represented and many others represented by only one or two participants, see Figure (Figure 5b ). Collecting both nationality and formative country data was key, as early cultural exposure strongly influences musical preferences, emotional interpretation, and listening habits. These sociocultural factors shape how individuals perceive and respond to music which could help interpret certain emotion patterns that arise in the data or explain variability [30, 31].

Gender identity included 48 female, 45 male, 2 non-binary participants and 3 left blank. The most common occupations were university students (32) and full-time employment (32), followed by part-time employment (14), self-employment (8),“other” (7), retired (3), at school (1), and full-time parent (1).

This study was approved by Queen Mary University of London Ethics Committee (QMERC20.565.DSEECS23.131). All participants provided informed consent prior to participation, and data was anonymised and stored securely according to university guidelines.

### Musical background and listening habits

To contextualize participants’ emotional responses to music, a subset of the Goldsmiths Musical Sophistication Index (Gold-MSI) was used [32]. Rather than the full scale, the subscales most relevant to emotion perception, namely Musical Training, Perceptual Abilities, Emotions, and Active Engagement, were selected (21 in total). Three additional questions were assessed; preferred genre, listening context (focused versus multitasking), and experience with digital audio workstations (DAWs), reflecting contemporary listening habits and factors particularly relevant for film-music perception.

Participants reported the amount of time they spend listening to music daily, selecting from fixed duration ranges for attentive (focused) and casual (background/multitasking) listening Results are summarised in Table 6b.

**Fig. 6:**
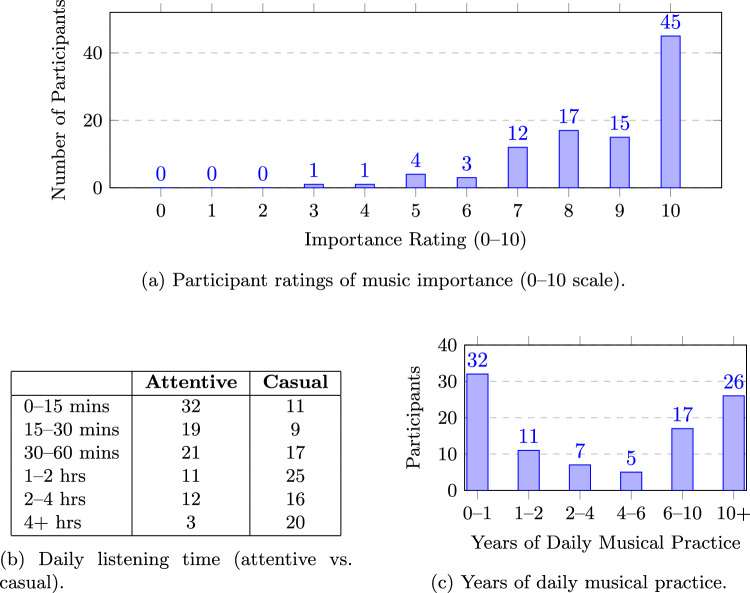
Participant musical engagement: (a) music importance ratings; (b) daily listening time; (c) years of musical practice.

The most common duration for attentive listening was 0-15 minutes (32 participants), while casual listening peaked at 1-2 hours (25). Casual listening was more common at longer durations, indicating frequent everyday engagement (see Table 6b ). Participants also reported years of daily instrumental or vocal practice, grouped into six ranges. Most had minimal experience (0-1 years, 32 participants), while 26 reported extensive experience (10+ years), showing a broad range of musical backgrounds (see Figure 6c ).

Participants reported 64 unique favourite genres, often combining sub-genres. These were manually grouped into broader categories based on stylistic similarity (e.g., techno and drum & bass classified under electronic) to simplify analysis. Genre preference can shape emotional interpretations and reveal personal listening biases. The most common genres were Rock (26), Electronic (18), Indie (18), Pop (16), and Classical (8). Music importance ratings (0-10) were strongly skewed high: 45 participants selected 10, and 32 selected 9 or 8; none rated below 3 (Figure 6a ). This suggests participants are highly engaged with music, supporting reliable annotations but indicating a bias toward musically invested listeners.

## Inter-annotator agreement

Inter-annotator agreement was calculated using Krippendorff’s alpha, a standard reliability metric used to measure agreement between annotators and suitable for sparse continuous data. The dataset is sparse and asynchronous because annotators freely placed V-A annotations over time, resulting in different annotators contributing varying numbers of annotations per excerpt. To compute alpha, mean valence and arousal values were calculated per annotator per excerpt and arranged into an annotator-by-excerpt matrix. Krippendorff’s alpha was then computed separately for valence and arousal using interval measurement level.

Agreement values were low, which is expected because annotators could freely place V-A annotations at any time and contribute varying numbers of annotations, making perfect alignment across annotators unlikely (Table 3). Additionally, Valence-Arousal annotation can be unintuitive for non-expert users, and emotional perception is subjective.

Agreement was therefore also analysed directly in the V-A space by measuring the spatial proximity of annotations. For each excerpt, pairwise Euclidean distances were computed between all annotations from different annotators. The mean pairwise distance was 0.66 (SD = 0.37), with a median of 0.61 (Table 3). Given that the maximum possible distance in the V-A space ($-1$ to 1 on both axes) is approximately 2.83, these results indicate that annotations cluster within a relatively small region of the V-A space.

**Table 3:** Inter-annotator agreement analysis. Krippendorff’s $\alpha$ measures classical rater agreement, while pairwise Euclidean distance measures spatial similarity between emotional annotations in valence–arousal space.

(a) Inter-annotator agreement using Krippendorff’s a	
**Krippendorff’s Alpha (IAA)**	
**Dimension**	$\alpha$
Valence	0.011
Arousal	0.126
(b) Spatial agreement between annotations in valence-arousal space.	
**Pairwise Euclidean Distance**	
**Statistic**	**Value**
Mean	0.663
Median	0.611
Standard Deviation	0.369

**Fig. 7:**
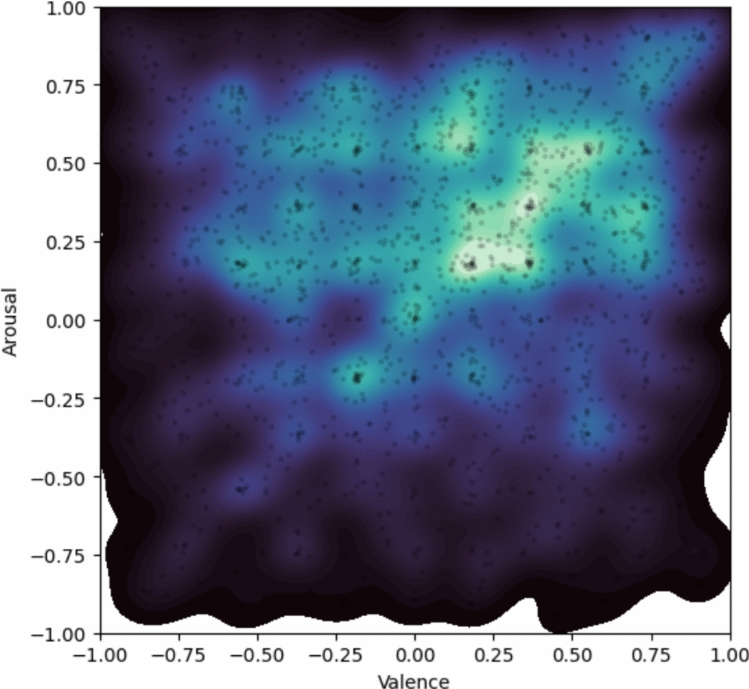
Kernel density estimate (KDE) showing the distribution of participants’ V-A coordinates in the valence-arousal space.

Figure 7 shows the distribution of annotations using a kernel density estimate (KDE). Lighter regions indicate higher annotation density, while darker regions indicate lower density. Individual annotation points are also shown. For visual clarity, (0, 0) annotations were excluded. Removing these points allowed lower-density emotional clusters to become more visible. The plot indicates that some regions of the V-A space have higher annotation densities (e.g., +A,+V quadrant).

## Musical feature extraction

Audio features were extracted using popular feature extraction toolboxes^1^. Features were extracted from 2-second windows, 1-second either side of the timestamp. A two-second window was selected as a balance between temporal precision and the need for musically interpretable feature extraction: segments shorter than two seconds risked insufficient information for reliable tonal or harmonic analysis, whereas longer windows (e.g., the full 15-second excerpt) would obscure rapid emotional shifts and conflate multiple perceived changes. The mean and variance of features (where appropriate) were extracted as a representation for those windows. In total, 78 features were extracted from each excerpt. These features can be grouped into several families: spectral and timbral features (e.g., spectral centroid mean, roughness mean), MFCCs and their derivatives, rhythmic and temporal descriptors (e.g., bpm, onset density), harmonic and tonal features (e.g., main key, tonnetz mean), dynamic/loudness features (e.g., rms db variance), and pitch/melodic descriptors (e.g., melodic range, vibrato extent).

To ensure all time-frames remained within the valid 0-15 s range, timestamps were first imputed where missing: single annotations were assigned the midpoint (7.5s), and multiple annotations were evenly spaced across the excerpt. Extraction windows were then defined as one second before and after each (original or imputed) timestamp and clipped at 0 and 15s to avoid extending beyond the excerpt boundaries.

## Baseline experiments

Four regression models were trained to predict valence and arousal from extracted audio features. Because annotations were placed freely in time, timestamps were grouped into 3-second windows to reduce temporal sparsity. Mean valence and arousal values within each window were calculated to form a single target observation.

78 audio features were extracted using the Librosa and Essentia toolboxes, representing spectral, rhythmic, and timbral descriptors. All features were standardised using z-score normalisation. Features with zero variance were removed using a variance threshold filter, and mutual information regression was used to estimate the relationship between each feature and the target variables. The top 40 features with the highest average mutual information scores were retained for model training, these can be found in the GitHub Repository.

The processed dataset was divided into training and testing subsets using an 80/20 split. Four regression approaches were evaluated: Random Forest, Light Gradient Boosting Machine (LightGBM), a Deep Neural Network (DNN), and a hybrid model. Random Forest used 300 trees, while LightGBM was trained with 2000 estimators, a learning rate of 0.01, and 80 leaves per tree. The DNN consisted of two fully connected layers (128 and 64 neurons with ReLU activation) with batch normalisation and dropout (0.3 and 0.2), followed by a linear output layer. Neural network models were trained using the Adam optimiser with mean squared error loss, and early stopping based on validation loss. The hybrid ensemble combined predictions from LightGBM (50%), Random Forest (20%), and DNN (30%).

Model performance was evaluated using Mean Absolute Error (MAE), Root Mean Squared Error (RMSE), and the coefficient of determination (R$^2$). In addition to standard regression metrics, a tolerance metric (±0.2) was used to measure the percentage of predictions where valence or arousal values fall within 0.2 of the ground-truth annotation.

Experiments were conducted on both the full dataset and a musician-only subset derived from the Gold-MSI questionnaire responses. The full dataset contains 1784 annotations from 98 participants across 422 excerpts, with an average of 4.2 annotations per excerpt (median 3). The musician-only subset contains 1045 annotations from 57 participants across 310 excerpts, averaging 3.4 annotations per excerpt. Most excerpts were annotated by 1-2 participants, highlighting the sparse and subjective nature of continuous affective ratings. Results are shown in Table 4.

**Table 4:** Baseline regression performance for predicting valence and arousal using extracted audio features. Results are shown for (a) all participants and (b) musician-only annotations. Using A Random Forest Regressor, LightGBM, Deep Neural Network and a Hybrid Model.

(a) Regression performance using all participant annotations.						
	**All Participants**					
**Model**	**Valence**			**Arousal**		
	R$^2$	MAE / RMSE	±0.2	R$^2$	MAE / RMSE	±0.2
RF	0.36	0.25 / 0.32	50.23%	0.30	0.24 / 0.32	53.73%
LightGBM	0.33	0.25 / 0.33	50.30%	0.26	0.24 / 0.33	53.58%
DNN	**0.42**	**0.24 / 0.31**	50.68%	**0.39**	**0.23 / 0.30**	52.66%
Hybrid	0.39	0.24 / 0.31	**51.90%**	0.34	0.23 / 0.31	**54.64%**
(b) Regression performance using musician-only annotations.						
	**Musicians Only**					
**Model**	**Valence**			**Arousal**		
	R$^2$	MAE / RMSE	±0.2	R$^2$	MAE / RMSE	±0.2
RF	0.37	0.24 / 0.31	53.34%	0.42	0.23 / 0.30	56.76%
LightGBM	0.31	0.25 / 0.33	53.63%	0.36	0.24 / 0.32	54.91%
DNN	0.34	0.25 / 0.32	50.07%	0.44	0.23 / 0.30	52.35%
Hybrid	**0.37**	**0.24 / 0.31**	**53.91%**	**0.44**	**0.22 / 0.30**	**58.04%**

Bold values indicate the highest score for each model in each instance (across the four evaluated instances)

The label “(a)” denotes results for the all_participants condition, while “(b)” denotes the musicians_only subset

## Usage

All CSV files, including features and metadata, are available on GitHub. The main file librosa_features_full _fme_dataset_MARCH_2026_NC.csv contains 1,784 rows with timestamps, V–A ratings, emotion sentences, familiarity scores, and audio features; participant demographics and GoldMSI responses are in fme-demographics-music-sophistication.csv and viewable interactively. Audio is not included due to copyright, but ISRC codes and a reference Spotify playlist are provided, as well as metadata in film-emotion-music-datasheet-275.csv. The original audio files were downloaded in MP3 format, with sample rates of 44.1 kHz or 48 kHz, and a nominal bit rate of 32 kbps. The sample rates are included in the metadata CSV file. Dataset access: https://github.com/rubycrocker/FME-24-dataset.

## Discussion

The FME-24 dataset offers a nuanced view of emotional responses to film music, capturing both arousal and valence over time. The annotation task was designed around the concept of “emotion change”, where participants marked transitions such as shifts between emotional states or noticeable increase/decrease of one emotion. The combination of V-A annotations and free-text descriptions allowed participants to express these changes with flexibility. Capturing real-time emotional dynamics in music remains inherently challenging, particularly when relying on subjective, continuous annotation paradigms.

The annotations capture emotion as a series of change-points rather than a continuous signal, reflecting how people naturally report noticeable shifts instead of constantly updating their feelings. The first annotation represents the participant’s first reported emotion, while the (0, 0) starting point is just a computational reference and does not imply a neutral state. This results in sparse annotations which vary in time across participants and are not aligned. Rather than forcing alignment, the data is treated as sparse emotional trajectories and handled using windowed aggregation, with initial analysis focusing on patterns in the V-A space. This allows meaningful trends to be identified despite variability.

Although Krippendorff’s alpha indicates low agreement, this should be interpreted in the context of the annotation design. The asynchronous nature of the task makes strict agreement difficult to achieve, meaning that low alpha does not necessarily imply unreliable annotations. The spatial analysis provides a more informative perspective: annotations cluster within relatively constrained regions of the valence-arousal space. This suggests that, despite differences in timing and annotation frequency, participants broadly agree on the general emotional character of the music.

The non-uniform distribution of annotations, particularly the higher density in regions such as high arousal and positive valence, indicates that certain emotional states are more frequently or more confidently identified. This has important implications for modelling, as it may introduce bias towards more densely represented regions of the affective space. Overall, these findings suggest that emotional annotations are better interpreted as probabilistic or region-based rather than precise point estimates, reflecting the inherently subjective and continuous nature of music-induced emotion.

Focusing on award-winning films ensures high musical quality, which supports re-use and style comparison research. However, generalizability is constrained by the curated sample; covering a broader range requires substantially more annotations and participants to make it reliable, prioritizing quality over quantity was necessary [33]. Although recognition selection provides an objective criteria beyond personal preference, it may not capture the full diversity of musical and cinematic expression. The film and music industries are known to be male-dominated and culturally skewed, meaning award recognition does not necessarily reflect broad ethical or demographic representativeness [34]; this was considered in selection, with efforts to include under-represented groups. Some compilation tracks carry extra-diegetic associations that may influence responses. Participant demographics also introduce bias: the majority were aged 18-35, predominantly Western, and English-speaking. While other attributes such as gender and occupation were more balanced, the dataset may still reflect culturally specific patterns of emotional perception.

The baseline models performed similarly across all four approaches, suggesting that improvements may be constrained by the dataset rather than the modeling approach. The dataset is relatively small and sparse, with most excerpts annotated by only 1-2 participants. This sparsity, along with the inherently subjective nature of emotional responses to music, may limit achievable predictive accuracy. The musician-only subset was evaluated to explore whether restricting annotations to participants with musical training improved predictive performance by reducing annotation variability. The tolerance metric (7) accounts for the fact that small numerical differences in continuous emotional ratings may still reflect perceptually similar emotional responses. Emotional responses vary across individuals, meaning that the same stimulus does not necessarily produce identical valence-arousal ratings [35]. Prior research also suggests that valence tends to exhibit greater variability than arousal, which may contribute to differences in predictive performance across dimensions [36].

Feature representation also plays a key role. Low- and mid-level audio features such as MFCCs, chroma coefficients, and timbral measures interact to produce complex perceptual qualities, and analysing them in isolation may obscure these relationships. For example, a single MFCC coefficient reveals little about timbre, but examining all coefficients together provides a fuller picture. While reducing the feature set can help mitigate overfitting in small, sparse datasets and reduce the risk of the curse of dimensionality, it may also limit the model’s ability to capture higher-level musical structure. Incorporating more informative composite or interaction-based features could therefore improve both predictive performance and interpretability.

## Limitations and future work

The study relied on a web-based annotation platform, and although participants were advised to use specific browsers and high-quality audio equipment, actual listening conditions could not be controlled. Variations in hardware, software, and environment may have affected both audio playback and annotation behaviour.

Metadata and ISRC codes were manually collected from multiple platforms, a process that was time-consuming and potentially error-prone. This constrained the overall scale of the dataset. Additionally, copyright restrictions prevent redistribution of the audio, limiting reproducibility and requiring future users to independently obtain the source material.

Participants could re-listen and adjust their annotations, which may have influenced responses compared to initial, instinctive reactions. This was intentional, as 15-second excerpts provide limited context for perceiving emotion on a single listen. The 15-second length represents a compromise between temporal precision and musically interpretable segments: shorter excerpts risk insufficient context, while longer excerpts increase cognitive load and make pinpointing change points harder, 15-second length is backed by other datasets (1). Although 2-second annotation windows allow fine-grained marking of perceived emotional shifts, participants typically indicated only 2-3 change points per excerpt. As a result, gradual or subtle emotional changes, such as; slow tension building in film scores, may not be fully captured. Also, Arousal and valence perception can vary on different timescales [25, 37]. Exploring longer excerpts or overlapping windows could help capture gradual emotional changes more effectively.

The use of fixed 3-second temporal clustering in the baseline models simplifies alignment across annotators, but may smooth over finer-grained or rapid emotional changes. Additionally, while mutual information-based feature selection identifies broadly informative predictors, it does not capture feature interactions or provide fully interpretable importance rankings, which could be explored in future work. Potential temporal misalignment between annotated excerpts and reconstructed audio (e.g., via Spotify/ISRC) may introduce minor discrepancies in feature-annotation correspondence, which should be considered when reusing the dataset.

Expanding the dataset to include more participants and a wider range of musical material would improve reliability and generalisability. In particular, increasing cultural and geographic diversity, as well as including more non-Western films, would support cross-cultural analysis of music-induced emotion.

Annotation methods could also be refined. Alternative approaches such as continuous annotation interfaces, lab-based studies, or group listening environments (cinema) could provide more consistent and temporally aligned data.

More advanced approaches in modelling, such as; transformer-based architectures or models incorporating temporal dependencies could better capture the dynamic nature of emotional responses. Transfer learning and subgroup analyses (e.g., based on GoldMSI responses) may further improve performance and provide deeper insights. In particular, one of the original motivations for creating this dataset was to compare emotional responses to original film scores versus compilation tracks. While this analysis has not yet been conducted, it would allow investigation of whether these subsets differ systematically, for example; due to familiarity, or differences in the compositional intent behind the music.

Finally, there is scope to develop emotion representations tailored specifically to film music. Analysis of free-text annotations suggests that participants’ language does not always align with standard valence-arousal models, highlighting the need for more domain-specific emotion frameworks and improved natural language processing techniques.

## Conclusions

The dataset is designed to capture dynamic emotional changes across film music. These time-stamps and V-A annotations enable researchers to examine how perceived emotion evolves moment-to-moment, supporting studies of the perceptual and cognitive processes underlying film music-emotion interactions. By integrating both quantitative and qualitative dimensions, FME-24 supports research across music psychology, film studies, and computational modelling, enabling applications in emotion recognition, soundtrack analysis, and the development of specialised analytical tools. Expanding the dataset with additional participant annotations and a broader range of audio tracks would strengthen reliability, improve generalisability, and support more robust machine-learning methods. At the time of writing, there are no immediate plans to release updated versions of FME-24; however, future expansions would be valuable and remain an open possibility. Overall, the dataset provides a foundation for studying emotion trajectories in film music and for developing new film-music-specific emotion models that reflect the relationship between musical structure, narrative and perceived emotion.

The authors declare that they have no competing financial or non-financial interests. This work was supported by UK Research and Innovation.

## Data Availability

The FME-24 dataset generated and analysed in this study is publicly available. All data supporting the findings of this study are provided within the manuscript and its Supplementary Information.

## Footnotes

1 Librosa, Essentia and Chordino
